# Response: “Commentary: Comparison of spike parameters from optically identified GABAergic and glutamatergic neurons in sparse cortical cultures”

**DOI:** 10.3389/fncel.2015.00224

**Published:** 2015-06-09

**Authors:** Keiko Weir, Oriane Blanquie, Werner Kilb, Heiko J. Luhmann, Anne Sinning

**Affiliations:** Institute of Physiology, University Medical Center of the Johannes Gutenberg UniversityMainz, Germany

**Keywords:** neuronal culture, multi-electrode array, imaging, interneurons, network activity, spike waveform

We are pleased to note that our publication “Comparison of spike parameters from optically identified GABAergic and glutamatergic neurons in sparse cortical cultures” by Weir et al. (2015) raised some discussion on the feasibility of solely electrophysiological discrimination of distinct neuronal subpopulations *in vitro*. We agree with Becchetti and Wanke (2015) that their report and our study on the same question were conducted with different technical approaches and that this may explain the observed differences between both studies. Although we obviously recorded a reduced spontaneous neuronal activity under our sparse culture conditions, these conditions were necessary to enable the unequivocal identification of single units in recordings with extracellular electrodes. Our combined approach of extracellular single unit recordings and cellular calcium imaging analyses in sparse neuronal cultures from GAD67-GFP transgenic mice clearly allowed the definite identification of inhibitory GAD67-positive interneurons and excitatory GAD67-negative neurons. The ratio of excitatory to inhibitory neurons and thus also the relative density of inhibitory connections was carefully assessed in our study and comparable to data from the cerebral cortex *in vivo*. For technical reasons this unambiguous assignment of a single neuron to one recording electrode is not possible at high cellular densities.

We performed additional experiments and now also analyzed Fano factors of spike counts recorded in culture medium and under different culture conditions (low density culture 15.6 ± 1.8 recorded neurons; medium density culture 54.3 ± 3.1 recorded neurons). Fano factors of these cultures were compared to those of clearly identified GFP-positive, inhibitory and GFP-negative, excitatory units published in our previous study (Weir et al., 2015). Fano factors were calculated for all units by dividing the variation by the average spike counts within a time window of 800 ms. Note, that Becchetti et al. previously used a time window of 6 s for calculation of Fano factors (Becchetti et al., 2012). However, our additional analyses revealed that this methodological difference in the bin width does not account for the different experimental outcome, since Fano factors calculated with 6 s and 800 ms bins were strongly correlated (Low density culture *R*^2^ = 0.596, *p* < 0.0001; High density culture *R*^2^ = 0.649, *p* < 0.0001).

As shown in Figure 1 below, all four experimental groups show a similar distribution of Fano factors and no significant differences in average Fano factor in different conditions (low density cultures 2.91 ± 0.16, *n* = 204 cells; medium dense cultures 2.6 ± 0.17, *n* = 152 cells; GFP-negative neurons 2.83 ± 0.3, *n* = 81 cells: GFP-positive interneurons 3.23 ± 1.03, *n* = 20 cells; One-Way ANOVA *R*^2^ = 0.005, *p* = 0.54). Single unit recordings at culture conditions with 1000 cells per mm^2^ as suggested by Becchetti and Wanke are not possible because spike sorting algorithms under this condition revealed a high failure rate.

**Figure 1:**
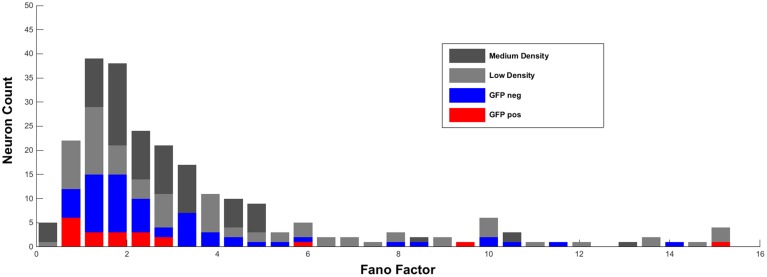
**Distribution of Fano factors (calculated as described in Weir et al., 2015) of single units in low and medium dense cultures recorded in culture medium**. For comparison, Fano factors of visually-identified GFP-positive and GFP-negative units recorded in ACSF are added.

Thus, on the basis of our new and additional experiments and analyses we cannot confirm that the Fano factor represents a sufficient parameter to reliably discriminate under *in vitro* conditions between excitatory and GABAergic inhibitory neurons.

However, we would like to point out that a number of previous studies (Barthó et al., 2004; Sirota et al., 2008; Sakata and Harris, 2009) and also our own analyses in adult rat cerebral cortex (Reyes-Puerta et al., 2014) were able to identify inhibitory interneurons recorded under *in vivo* conditions on the basis of their extracellular firing properties. Therefore, it cannot be excluded that a more immature functional state of neurons cultured under *in vitro* conditions hamper the identification of inhibitory interneurons on the basis of the extracellular spiking properties.

## Conflict of interest statement

The authors declare that the research was conducted in the absence of any commercial or financial relationships that could be construed as a potential conflict of interest.
